# What are Romantic Relationships Good for? An Explorative Analysis of the Perceived Benefits of Being in a Relationship

**DOI:** 10.1177/14747049231210245

**Published:** 2023-10-31

**Authors:** Menelaos Apostolou, Christoforos Christoforou, Timo Juhani Lajunen

**Affiliations:** 1 Department of Social Sciences, 121343 University of Nicosia, Nicosia, Cyprus; 2 Department of Psychology, 8018 Norwegian University of Science and Technology (NTNU), Trondheim, Norway

**Keywords:** intimate relationships, benefits of intimate relationships, mating, long-term mating

## Abstract

Forming long-term intimate relationships is a human universal, with most people across different times and cultures doing so. Such relationships should be associated with important benefits otherwise individuals would not engage in them, with the current research aiming to identify what people consider as beneficial in a long-term intimate relationship. More specifically, Study 1 employed qualitative research methods in a sample of 221 Greek-speaking participants, and identified 82 perceived benefits. Study 2 employed quantitative research methods in a sample of 545 Greek-speaking participants, and classified these benefits into 10 broad factors and two broader domains. Experiencing positive emotions, including love and passion, as well as having someone to provide support and do things together, were considered among the most important benefits. Although there were a few significant differences, the evaluations of the perceived benefits of intimate relationships were generally consistent across participants of different sex, age, and relationship status.

## Introduction

Across different times and different cultures, people form long-term intimate relationships (Brown, 1991; Fisher, 2017), which suggests that they are accompanied by important benefits. The purpose of the current research is to examine what individuals consider as beneficial in being in an intimate relationship. These benefits can be better understood within an evolutionary theoretical framework that will be discussed next.

### Why People Form Long-Term Relationships

Human mating is strategic in the sense that people employ specific strategies in diverting their resources toward achieving specific mating goals (Gangestad & Simpson, 2000). Children require considerable, reliable, and prolonged parental investment in order to reach sexual maturity and become themselves able to procreate (Hawkes et al., 1989; Kim et al., 2012). This fact favors the evolution of a long-term mating strategy, where individuals look for mates who would stay with them in the long term, and would provide for their children (Buss, 2016; Gangestad & Simpson, 2000). Moreover, people require reliable assistance, support, and protection from others in order to meet the challenges of survival. This was particularly the case in ancestral human societies, where there were few if any social protection systems, and individuals relied heavily on others, especially during difficult times. Furthermore, having different casual partners increases the risk of getting a sexually transmitted disease, some of which were fatal in the ancestral context (Fletcher et al., 2015; Kokko et al., 2002). The need for support and protection would also favor a long-term mating strategy, where people would aim to attract mates who would provide assistance and support for them in the long run (Apostolou, 2021; Buss, 2016; for further discussion on the evolution of monogamy see Klug, 2018; Schacht & Kramer, 2019).

These selection pressures have resulted in long-term mating to be the most prevalent mating strategy, with different lines of evidence supporting this prediction. In particular, sexual dimorphism exists within a species when the sexes differ in size or appearance (Andersson, 1994), with limited sexual dimorphism in body size being associated with monogamy (Harcourt et al., 1981). Modern humans express limited differences in body size by sex compared to closely related promiscuous and polygynous species. For example, human males are on average 15% heavier than women and are closer to monogamous gibbons where males are 7% heavier than females, than to promiscuous gorillas where males are 100% heavier than females (Dixson, 2009; Plavcan & van Schaik, 1992; Willner, 1989; see also Kappeler, 2014). Moreover, the high occurrence of a long-term mating strategy is reflected in the institution of marriage, which is a human universal; that is, all past and present societies practice marriage, with the majority of people eventually getting married (Brown, 1991; Coontz, 2005). In the same vein, one recent study employed a sample of 6,273 Greek-speaking participants and found that about 83%, aspired to form initially or eventually a relationship that would last a lifetime (Apostolou, 2021).

#### The Benefits of Being in an Intimate Relationship

The above theoretical framework indicates that one major survival and reproductive or fitness benefit of long-term relationships is to have someone around who would provide reliable support and protection (H_1_). That is, people would consider it beneficial to enter into an intimate relationship, because by doing so, they would get an intimate partner who would provide them and their children with reliable support, and would be there for them in times of need. The fitness benefits of long-term intimate relationships would favor the evolution of mechanisms such as emotions (Tooby & Cosmides, 2008) that would motivate people to attract and retain long-term intimate partners. In particular, negative emotions such as loneliness, sadness, and jealousy, would trigger when individuals do not have an intimate partner, while positive emotions such as happiness and joy, would trigger when individuals manage to secure an intimate partner (Apostolou et al., 2019). Accordingly, we hypothesize that one benefit of being in an intimate relationship would be experiencing positive and avoid experiencing negative emotions (H_2_).

In the same vein, sexual desire mechanisms would motivate people to seek intimate partners in order to have regular sexual contact (Meston & Buss, 2007; Toates, 2014). Accordingly, we hypothesize, that people would consider receiving sexual satisfaction and having regular sex to be another benefit of being in an intimate relationship (H_3_). Emotional adaptations are proximate mechanisms that serve the ultimate goal of propagating the genes that code for their ontogenetic development. One way to achieve this goal is to generate emotional responses that motivate the formation of long-term intimate relationships, which have important fitness advantages. Positive emotions and sexual satisfaction are indirect fitness benefits, that is, they do not directly increase fitness the same way as receiving support from a partner does, but do so indirectly by motivating people to form lasting intimate relationships in order to enjoy them. The existing literature provides some support for these hypotheses.

### Current Literature

Several studies have found that marriage is associated with benefits including physical health and longevity, mental health and happiness, economic well-being, children performing better (i.e., children raised by their own married parents do better across a range of outcomes than children in different living arrangements), and higher sexual satisfaction (for a review see Waite & Lehrer, 2003; Wilcox, 2011). To use some more specific examples, a meta-analysis involving 34 studies with more than two million participants, found that married people were less likely to suffer from cardiovascular diseases (Wong et al., 2018). Another meta-analysis of 15 studies and more than 800,000 participants, found that being married was associated with a reduced risk of dementia than being widowed or lifelong single (Sommerlad et al., 2017).

A different line of research investigated the effect of being in different categories of relationship status on positive emotions and life satisfaction. More specifically, Apostolou et al. (2019) employed a sample of 735 Greek-speaking participants and found that people who were in a relationship or married, experienced fewer negative and more positive emotions as well as higher life satisfaction than those who were single. A replication study performed during the COVID epidemic found similar results (Apostolou & Kagialis, 2020). In the same vein, Van De Velde et al. (2010) found that being single was a large risk factor for high levels of depression in men. A recent study compared men who identified as incels (i.e., men who forge their sense of identity around a perceived inability to form intimate relationships) with non-incels, and found that the former had higher levels of depression, anxiety and loneliness, and lower levels of life satisfaction (Costello et al., 2022).

What people consider beneficial in a relationship would correlate with what motivates them to keep one, with a recent study investigating the motives for keeping an intimate relationship (Apostolou, 2022). More specifically, using a combination of qualitative research methods in a sample of 131 Greek-speaking participants, it identified 58 reasons that motivated individuals to keep their intimate relationship. Subsequently, using quantitative research methods in a sample of 789 Greek-speaking participants who were in an intimate relationship, it classified these reasons into nine broad factors. Having a supporting and compatible partner with whom one shares similar goals, and with whom one has good sex and a strong emotional attachment, were rated among the most important factors motivating participants to keep their relationship.

### The Current Study

Existing literature has identified several benefits of being in an intimate relationship, such as better health and experiencing positive emotions. However, these findings may not necessary reflect what people consider beneficial in being in an intimate relationship. For instance, because studies have found that being mated is associated with higher life expectancy, this does not mean that people would actually consider longevity a benefit of intimate relationships. To the best of our knowledge, there has not been any study that has specifically attempted to identify what people perceive as beneficial in being in an intimate relationship, which is the purpose of the present work. This endeavor is worth pursuing because, by studying what people perceive as beneficial in being in a lasting intimate relationship, we can better understand the motivation for forming (or not forming) and keeping one.

In more detail, Study 1 employed qualitative research methods in order to create an inclusive list of what people consider beneficial in being in a long-term intimate relationship, and Study 2 employed quantitative research methods in order to classify these benefits into broader categories. We hypothesize that these categories would reflect three main benefits namely, having someone around to provide support and protection, enjoying positive emotions, and having regular sex. Nonetheless, our study is explorative, as the complexity of the phenomenon and the limited research in the area, prevents us from forming hypotheses for all possible benefits of being in an intimate relationship. In addition, in order to examine whether men and women and people in different age and relationship status groups viewed the benefits of intimate relationships differently, we examined sex, age, and relationship status effects, without however making directional hypotheses.

## Study 1

### Method

#### Participants

The study was conducted at a private university in the Republic of Cyprus and has received ethics approval from the institution's ethics board. Participants were recruited by promoting the link of the study on social media (i.e., Facebook and Instagram), and by forwarding it to students and colleagues, asking them to forward it further. The only requirement for participation was to be at least 18 years of age. Participation was on a volunteer basis, and no monetary or other incentives were given. Overall, 239 individuals took part in the study. Yet, in order to increase validity, we excluded participants who indicated that they were currently single and had never been in a relationship before. In effect, we analyzed the responses from a sample of 221 Greek-speaking individuals (120 women, 101 men). The mean age of women was 27.8 (*SD* = 10.2), and the mean age of men was 28.2 (*SD* = 11.1). With respect to the relationship status, 24.2% of the participants indicated that they were involuntarily single, 23.7% in a relationship, 17.6% voluntarily single, 13.1% in single-between relationships, 12.3% married, and 9.1% indicated their relationship status as “other.”

### Materials

The study was in Greek, run online, and was created using Google Forms. It had two parts. In the first part, participants were asked the following: “Write down some benefits that you think those who are in a long-term intimate relationship enjoy,” and they were given space to record their answers. In the second part, demographic information was collected, including sex, age, and relationship status. Participants were also asked whether they had been in an intimate relationship in the past (Yes/No).

## Analysis and Results

For the purpose of analyzing our data, we recruited two independent graduate students (a man and a woman). Each research assistant was asked to go through the responses and prepare a list of benefits associated with being in an intimate relationship. The assistants were instructed to eliminate answers with unclear or vague wording. After processing about 30% of the responses, they discussed and compared their respective lists of benefits, and then moved on to process the remaining responses. Two lists of benefits, one from each assistant, were produced and were compared with each other. Agreement was found for most of the items. In cases where there was no complete overlap, the authors were consulted, and eventually, all the parties involved agreed to a final list of benefits. In total, 82 benefits were identified and are presented in Table 1.

**Table 1.** The of Intimate Relationships Identified in Study 1 and Their Classification into Broader Categories in Study 2.

Benefits items	Factor loadings	Cronbach's α
**Support**		
Help in difficult times	.571	.96
Psychological support	.565	
I have someone I can count on	.561	
I have someone to share thoughts/feelings/concerns/worries	.547	
I have someone to support me	.517	
I have someone who understands me	.501	
Support in the realization of my goals	.462	
Feeling of security	.441	
I have someone to be there for me	.440	
I have someone I trust	.393	
I have someone to listen to me	.379	
I have someone to protect me	.351	
I have someone who accepts me as I am	.329	
I have someone who appreciates me	.314	
I have someone to give me well-intentioned advice	.312	
I have someone to inspire me	.305	
I have someone with whom I am very close	.297	
**Social acceptance**		.82
Less pressure from society/parents/friends to have a relationship	.745	
I do not need to spend time and money looking for an intimate partner	.661	
I am more socially acceptable	.640	
I can focus on other things besides looking for a partner	.518	
I do not have to put a lot of energy into taking care of my appearance	.416	
Expanding my social circle	.324	
I have the prospect of having a family	.213	
**Sexual satisfaction**		.87
Sexual satisfaction	.861	
Good sex	.838	
I have someone to meet my sexual needs	.805	
Regular sex	.786	
Easier sex	.611	
Sex with someone I am emotionally involved with	.290	
**Have someone to keep me company and to do things together**		.88
I have someone to go out with	−.607	
Have someone to keep me company	−.532	
I have someone to go on trips with	−.528	
I have someone to talk to	−.488	
I have someone to do things with	−.456	
I have someone to talk to at any time	−.351	
I do not feel lonely	−.305	
I can discover new interests and hobbies with my partner	−.279	
Enthusiasm	−.251	
**Positive emotions**		.94
Positive emotions	.528	
Good mood	.486	
Joy	.478	
I am experiencing happy moments	.451	
Beautiful moments	.411	
Less stress and anxiety	.389	
Optimism	.382	
Warmth	.367	
Emotional fullness	.341	
The feeling of having someone to care about me	.335	
Creating beautiful memories	.328	
Beautiful words	.320	
More confidence	.278	
I have someone to complete me	.248	
Feeling of fulfillment	.218	
**Give and receive care**		.86
I have someone to take care of	−.552	
Feeling that someone needs me	−.462	
I have something to give meaning to my life	−.440	
I am important to someone	−.418	
I have someone to take care of me	−.345	
A sense of belonging somewhere	−.271	
**Safe sex**		.75
Safer sex	−.653	
Lower chance of getting a sexually transmitted disease	−.633	
I have a steady romantic partner	−.356	
Better health	−.338	
**Love and passion**		.94
Love	.682	
I have someone to hug me	.663	
Moments of tenderness	.588	
Affection	.547	
I have someone to hold me when I am sleeping	.546	
Companionship	.416	
I have someone to love	.373	
I have someone to love me	.362	
Passion	.362	
I have someone to share beautiful moments with	.329	
Deeper connection with someone	.326	
I have someone to share dreams and desires with	.273	
**Sharing expenses**		.79
Sharing expenses	−.894	
Economic support	−.762	
Sharing obligations	−.617	
Eat better	−.286	
**Stability**		.59
I have something certain	−.464	
Stability	−.291	

## Study 2

### Method

#### Participants

In order to recruit participants, we employed the same method as in Study 1. Overall, 583 individuals took part, but in order to increase validity, we excluded the answers of those who indicated that they were currently single and had never been in a relationship before. In total, 545 (299 women and 246 men) Greek-speaking individuals took part. The mean age of women was 30.6 (*SD* = 11.2) and the mean age of men was 32.3 (*SD* = 12.1). In addition, 38.2% of the participants indicated that they were in an intimate relationship, 20.0% single without wanting to be so, 14.5% between relationships, 12.7% married, 7.0% single by choice, and 7.7% indicate their relationship status as “other.”

### Materials

The survey was in Greek, run online, was designed using Google Forms, and consisted of two parts. In the first part, participants were given the following scenario: “Below, you will find a number of possible benefits of being in an intimate relationship. Please rate how important these benefits are for you.” Subsequently, participants were given the 82 benefits identified in Study 1, to rate on the following five-point Likert scale: 1 – Not at all important, 5 – Very important. The order of presentation of the questions was randomized across participants. In the second part, demographic information was collected, including sex, age, relationship status, and past relationship experience.

### Data Analysis

In order to classify the 82 benefits identified in Study 1 into broader categories, we applied principal components analysis, using direct oblimin as the rotation method. In order to decide how many factors to retain, we employed the Kaiser criterion that is, we retained all factors with an eigenvalue equal to or greater to one. In order to identify significant effects, we performed a series of MANCOVA tests on each extracted factor. More specifically, the items that loaded on a given factor were entered as the dependent variables, the sex was entered as the categorical independent variable, and the age as the continuous independent variable. We also aimed to examine whether there was a difference in the scores between participants who were in an intimate relationship and participants who were single. Accordingly, we created a new variable with three categories (i.e., single, in an intimate relationship, other), which was also entered as an independent categorical independent variable. In order to do so, we collapsed all the singlehood categories into one (i.e., single), the “in a relationship” and the “married” into one (i.e., in an intimate relationship), and we retained the “other” category.

## Results

### Factor Structure

In total, 10 factors were extracted and are presented in Table 1. Internal consistency (Cronbach's α) ranged from .59 to .96. The first factor to emerge was “Support,” where participants considered it beneficial to have an intimate partner who would help them in difficult times, would understand them, would support them in achieving their goals, would protect them, would advise them, and would be there for them. In the “Social acceptance” factor, by being in a relationship, people would be more socially acceptable and would experience less pressure from their family and friends to form a relationship. Another benefit that participants indicated was “Sexual satisfaction,” which involved better and more regular sex. Moreover, in the “Have someone to keep me company and to do things together” benefit, people considered it beneficial to have around someone to keep them company so they did not feel alone, as well as to go out and travel together.

Moving on, participants indicated that one benefit of intimate relationships was to experience “Positive emotions,” including joy, optimism, and warmth. In the “Give and receive care” factor, one benefit people indicated was to have someone to care for as well as to have someone to care for them. The “Safe sex” and fewer chances to contract a sexually transmitted disease, were also considered beneficial in an intimate relationship. In addition, experiencing “Love and passion” factor, as well as companionship and affection, were indicated as benefits. Furthermore, in the “Sharing expenses” factor, participants indicated that one benefit of being in an intimate relationship was to have someone to share expenses and obligations. Lastly, participants indicated “Stability” to be another benefit of being in an intimate relationship.

Means and standard deviations were calculated for each factor and are placed in a hierarchical order in Table 2. At the top of the hierarchy was the “Love and passion,” followed by the “Positive emotions,” and the “Support” factors. At the bottom of the hierarchy, were the “Sharing expenses” and the “Social acceptance” factors. Moreover, for each factor, we estimated the percentage of participants who gave mean scores above “3.” Given the scale used, these percentages would tell us how many participants considered each benefit important. From Table 2, we can see that the highest percentage was for the “Love and passion” factor (95.5%), and the lowest one was for the “Social acceptance” factor (36.7%).

**Table 2.** Mean Scores, Sex, Age, and Relationship Status Effects in Study 2.

Factors		Overall	Women	Men	Sex		Age		Relationship status	
	%	Mean (*SD*)	Mean (*SD*)	Mean (*SD*)	*p-*value	η_p_*^2^*	*p-*value	η_p_*^2^*	*p-*value	η_p_*^2^*
Love and passion	95.5	4.46 (0.69)	4.56 (0.57)	4.32 (0.80)	.004	.056	.032	.044	.677	.020
Positive emotions	94.3	4.25 (0.74)	4.36 (0.65)	4.12 (0.82)	.011	.059	(-) .001	.075	.270	.034
Support	92.9	4.23 (0.78)	4.36 (0.70)	4.07 (0.84)	.009	.066	.049	.055	.001	.067
Have someone to keep me company and to do things together	88.4	3.97 (0.79)	4.05 (0.75)	3.87 (0.84)	.055	.032	.173	.025	.023	.032
Sexual satisfaction	85.9	3.97 (0.88)	4.08 (0.80)	3.83 (0.96)	.067	.023	.033	.027	.013	.025
Give and receive care	78.3	3.80 (0.95)	3.84 (0.95)	3.74 (0.96)	<.001	.095	(-) <.001	.050	.177	.016
Safe sex	77.8	3.77 (0.97)	3.94 (0.87)	3.56 (1.04)	.148	.013	.209	.012	.143	.012
Stability	62.4	3.59 (1.05)	3.65 (1.03)	3.52 (1.08)	.205	.006	.332	.004	.283	.005
Sharing expenses	41.8	2.85 (1.04)	2.96 (1.04)	2.71 (1.03)	.020	.023	.011	.026	.225	.010
Social acceptance	36.7	2.73 (0.95)	2.74 (0.97)	2.72 (0.92)	.004	.041	(+) .006	.039	.006	.030

*Note.* The signs in parenthesis indicate the direction of the relationship. The “%” column reports the percentage of participants who had a mean score above “3” in each factor.

In order to classify the identified factors into broader domains, we performed second-order factor analysis by applying principal axis factoring using the direct oblimin as the rotation method on the 10 extracted factors. The results indicated a two-domain solution. As we can see from Table 3, the first domain reflected benefits which were intrinsic to the relationship such as experiencing love and passion, and the second domain reflected benefits which were extrinsic to the relationship such as gaining social acceptance.

**Table 3.** The Extracted Domains in Study 2.

Domain factor	Factor loadings
**Intrinsic benefits**	
Love and passion	1.027
Positive emotions	.958
Support	.918
Have someone to keep me company and to do things together	.803
Give and receive care	.685
Sexual satisfaction	.362
**Extrinsic benefits**	
Social acceptance	.903
Sharing expenses	.864
Safe sex	.697
Stability	.531

### Significant Sex, Age, and Relationship Status Effects

In total, 10 MANCOVA tests were performed. Thus, in order to avoid the problem of alpha inflation, Bonferroni correction could be applied, setting the significance level to .005 (.050/10). As we can see from Table 2, significant sex differences were found for the “Love and passion,” the “Give and receive care,” and the “Social acceptance” factors, with women giving higher scores than men. For the rest of the factors, although the differences were not statistically significant, women gave also higher scores than men. Significant age effects were found for the “Positive emotions,” the “Give and receive care,” and the “Social acceptance” factors. For the first two, the coefficient was negative, indicating that older participants gave lower ratings than younger ones, while for the last factor, the coefficient was positive, indicating that older participants gave higher scores than younger ones. The relationship status was significant only for the “Support” factor, where people who were in an intimate relationship gave higher scores (*M* = 4.32, *SD* = 0.75) than those who were single (*M* = 4.14, *SD* = 0.80) or who have indicated their marital status as “other” (*M* = 4.32, *SD* = 0.73).

## Discussion

In the current research, we identified 82 possible benefits of being in a long-term intimate relationship, which were classified into 10 categories reflecting broader benefits, which in turn, were classified into two domains reflecting intrinsic and extrinsic benefits. Among the most important ones, were experiencing positive emotions, including love and passion, as well as having someone to provide support and to do things together. Although there were some significant differences, the evaluations of what was considered beneficial in intimate relationships were generally consistent across participants of different sex, age, and relationship status categories.

As we originally predicted, people considered having someone to provide them with “Support,” as a crucial benefit of being in an intimate relationship. This factor was rated third in importance, with about 93% of participants considering it beneficial. The “Give and receive care” factor, found in the middle of the hierarchy of importance, is to some degree consistent with this argument, as participants considered it beneficial to have someone to look after them. Likewise, the “Sharing expenses” factor, with participants indicating as a benefit of a relationship to have someone to share expenses and provide financial support, is also consistent with this prediction. Yet, this factor was second from the bottom in the hierarchy of benefits, with about 42% of the participants considering it important. In our hypothesis, people would consider the support they receive from their partners beneficial also for their children. Nevertheless, participants did not specifically reported that one possible reason being that they used the term “support” more broadly, and inclusive of supporting one's children.

Also consistent with our original hypothesis, participants indicated experiencing “Positive emotions,” and “Love and passion” in particular, as benefits of being in an intimate relationship. These two factors topped the hierarchy of importance, with more than 95% considering “Love and passion,” and more than 94% considering “Positive emotions,” as important benefits of being in an intimate relationship. Nevertheless, there was no specific factor indicating the avoidance of negative emotions as a benefit of intimate relationships. Yet, the “Have someone to keep me company and to do things together” factor, had such a facet: Participants indicated as beneficial of a relationship to have someone to keep them company so that they do not feel lonely. Furthermore, as we originally predicted, receiving “Sexual satisfaction,” involving regular and easier sex, emerged as benefit of being in an intimate relationship. This factor was found at the middle of the hierarchy of importance, with about 86% of the participants rating it as such.

Several factors emerged, which we have not originally predicted. To begin with, participants indicated the “Have someone to keep me company and to do things together” to be an important benefit of intimate relationships. People have a basic need to receive social input that is, to have around them others with whom they could interact, share their thoughts, and discuss what troubles them (Ristau, 2011). An intimate partner can to some degree fulfill this need, and people acknowledge it in the benefits composing this factor. This need is also satisfied by friends, so it is not surprising that, when people enter into a relationship, they tend to lose friends that is, their partners substitute some of their friendships (Hruschka, 2010). In addition, this factor indicated another benefit of being in an intimate relationship, namely people having a need to care for someone, and an intimate relationship gives them the opportunity to satisfy it.

Moreover, people indicated “Safe sex” to be another important benefit of being in an intimate relationship. This factor probably reflects increasing awareness of the causal link between having sex with different partners and the increase in the probability to contract a sexual transmitted disease, as well as the health risks associated with these diseases. For instance, it has been shown that, by increasing the costs of multiple mating, sexually transmitted diseases have favored relatively monogamous mating strategies (Kokko et al., 2002). In addition, people who are not mated, frequently experience social pressures to find a partner and have a family. In preindustrial societies, this pressure is stronger and more direct, taking the form of arranged marriage (Apostolou, 2007, 2010), while in post-industrial societies it takes the form of psychological manipulation (Apostolou & Papageorgi, 2014). Some scholars have argued that single people are actively discriminated, and call this singlism (DePaulo, 2006). These social pressures would be relaxed once people enter in an intimate relationship, which explains the emergence of the “Social acceptance” factor.

Female participants tended to rate the benefits of intimate relationships higher than male participants, with this tendency passing the significance level in three instances. The largest sex difference was over the “Give and receive care,” followed by the “Love and passion” factor. This finding could be interpreted to mean that men consider being in a long-term intimate relationship less beneficial than women. One possible reason is that, by virtue of not having to bear the burden of pregnancy, men can receive more reproductive benefits from short-term intimate relationships than women (Buss & Schmitt, 1993), and these benefits may enable them to discount the benefits of long-term mating.

Significant age effects were found for the “Positive emotions” and the “Give and receive care” factors, with older giving lower scores than younger participants. These findings may be interpreted to mean that emotional responses to intimate relationships as well as the need to give and receive care, are stronger in younger people. Moreover, for the “Social acceptance factor,” the age effect was very close to the Bonferroni-adjusted significance level, with older participants giving higher scores than younger ones. One reason could be that, as people get older and remain single, their friends and family worry more that they will remain unmated forever, and increase thus, their pressure on them to get an intimate partner. In effect, older people would find it more beneficial to be in an intimate relationship in order to gain social acceptance. Moving on, people who were in an intimate relationship rated the “Support” factor higher than people who were single, one possible reason being that, the support they received from their partners, made a noticeable difference in their lives (see also Bedair et al., 2020; Golestaneh, 2022). In general however, for most factors, there were no significant main effects of sex, age, and relationship status, indicating that men and women, older and younger, and mated and unmated individuals generally agreed on what is beneficial in an intimate relationship.

The observed domain structure indicates that, when people reflect on what is beneficial in a long-term intimate relationship, they consider primarily intrinsic benefits (the factors which loaded in this domain had the highest means), such as experiencing positive emotions, and secondary, the extrinsic benefits of it, such as relaxing social pressures. The “Love and passion” and the “Positive emotions” factors had the highest loading on the “Intrinsic benefits” domain, and the highest means among the identified factors. It is reasonable to assume that, what people consider beneficial in an intimate relationship, would motivate them to enter in one. In this respect, our findings suggest that people would be motivated to form a long-term intimate relationship primarily in order to experience positive emotions such as love (regarding the importance of love at the beginning of the relationship see Watkins et al., 2022). This is consistent with the evolutionary theoretical framework, in which emotions are evolved adaptations that motivate behavior—in this case, forming intimate relationships, which increase reproductive success (Buss, 2019; Fisher, 2000; Fisher et al., 2016; Gonzaga & Haselton, 2008).

What is considered beneficial in an intimate relationship, depends on the traits and circumstances of the individual. For instance, people who are well-off, may not consider a particularly beneficial of a relationship to have someone to share expenses, with the opposite being true for people who face financial difficulties. People who have a more independent personality, may value factors such as “Support” and “Have someone to keep me company and to do things together” less than people who have a more dependent personality. People who score high in agreeableness and enjoy helping others may value more the “Give and receive care” factor than low scorers. Accordingly, future research needs to identify which individual differences predict how people value the different benefits of being in an intimate relationship. Such endeavor would enable us to better understand how individual differences relate to relationship formation as well as to relationship quality (see Štěrbová et al., 2021).

How people value the different benefits of being in an intimate relationship would be also affected by cultural variables. For instance, in the Greek cultural setting, parents are proactive in motivating their children to enter in an intimate relationship, which suggests that, in this context, the “Social acceptance” benefit would be considered more important than in other cultural contexts where parents are less involved in mate choice. Similarly, people living in countries where there is only limited awareness of sexual transmitted diseases may consider the “Safe sex” benefit as less important than people living in countries where there is higher awareness. Thus, cross-cultural research is necessary in order to identify how cultural variables affect variation in how people assess the different benefits of being in an intimate relationship (see Karandashev, 2015, 2016; Schmitt, 2006).

Our study examined the perceived benefits of being in an intimate relationship, which do not necessary completely overlap with the actual benefits of being in an intimate relationship. For instance, studies consistently find that being in an intimate relationship is associated with important health outcomes (e.g., Wong et al., 2018); yet, in the current research, we did not find any health-related factors. Similarly, we employed an evolutionary perspective to guide us on the benefits of being in an intimate relationship. One such benefit is increasing one's chances of survival by having a partner to provide support and protection. Accordingly, we found that people perceived support to be an important benefit of being in an intimate relationship. Still, the proximate perceived benefits do not need to align with ultimate adaptive benefits (for a discussion on the differences between proximate and ultimate evolutionary functions see Wouters, 2003, 2013). Yet, evolutionary theory can guide us on the proximate mechanisms that motivate people to form intimate relationships in order to reap them. In particular, we have argued that emotions serve this purpose, and we have found that experiencing positive emotions such as love, were among the most important perceived benefits of being in a relationship. People form intimate relationships not because there are important fitness benefits in doing so, but because they perceive them as beneficial, and they do so because they have evolved mechanisms such as emotions which serve the ultimate purpose of motivating fitness-increasing behavior. Thus, the identified perceived benefits of being in an intimate relationship can enable us to better understand the motivation for forming one.

Furthermore, we have argued that having a long-term intimate partner has been associated with important benefits during human evolutionary time; yet, because modern conditions are different from ancestral ones, these benefits are likely to be different (see also Goetz et al., 2019). For instance, in contemporary post-industrial societies, there are protection and welfare institutions, including the police, unemployment benefits, social welfare benefits, and so on, which turn the support from a partner less important than in ancestral human societies where these institutions were not present. Having an intimate partner, at least for a while, is still necessary for having children; yet, the increasing use of sperm and egg donors, and surrogate mothers may also turn having an intimate partner less important for procreation (see also Fletcher et al., 2015). Similarly, modern medicine can deal effectively with most sexually transmitted diseases. Evolved mechanisms do not produce rigid responses, but adapt to environmental conditions, which means that, if the benefits of being in a long-term relationship decrease, so the motivation to enter in one decreases as well (for further discussion on motivation see Kenrick et al., 2010; Pick et al., 2022). In turn, this decrease in motivation could possibly account for increasing rates of divorce (Kennedy & Ruggles, 2014), and the high occurrence of singlehood (Apostolou et al., 2023).

On the other hand, it could be counterargued that, in contrast to ancestral human societies, people today live in large cities, surrounded by people they do not know and with whom they are not related. Accordingly, a long-term partner could be a valuable source for receiving social input, as well as support with everyday challenges. Similarly, although it is true that modern technology gives the opportunity of having a child without having an intimate partner, due to the high cost involved, access to this technology is beyond the reach of most people. Moreover, although welfare systems can support one-parent families, in most countries such support is far from adequate to cover a family's needs (Glass et al., 2016). In addition, medical breakthroughs have turn sexually transmitted diseases not lethal yet, some like HIV are difficult and expensive to treat, and can still cause considerable damage. Accordingly, being in a long-lasting relationship has important benefits in contemporary societies as well. Our findings are consistent with this argument as participants rated most of the benefits highly. For example, about 93% rated the “Support” as an important benefit of being in an intimate relationship.

One limitation of the current work is that it was based on non-probability samples, so its findings may not readily generalize to the population. Additionally, we employed self-report instruments which have several limitations, including participants not giving accurate answers. Moreover, our study was confined to the Greek cultural context, and its findings may not apply to other cultural settings. In addition, we focused on long-term intimate relationships; yet, forming short-term intimate relationships, is also part of the repertoire of human mating (Buss & Schmitt, 1993), and what people consider beneficial in these relationships needs to be also investigated. Furthermore, different people may have different definitions of what constitutes a long-term relationship. For instance, some may consider three months to be a long-term relationship, while others may consider five years as a minimum for a relationship to be considered long-term. Thus, future research needs to control how participants perceive the length of and the commitment to the relationship. Additionally, the current study focused exclusively on the benefits, but intimate relationships have also costs, that future research needs to examine.

Forming lasting intimate relationships constitutes a central aspect of human behavior. In the current research, we identified several perceived benefits of intimate relationships. Future studies that would employ different and more diverse samples, would enable us to better understand what people consider beneficial in being in a long-term intimate relationship.
